# Distraction sinking and fossilized coleoid predatory behaviour from the German Early Jurassic

**DOI:** 10.1186/s13358-021-00218-y

**Published:** 2021-03-16

**Authors:** Christian Klug, Günter Schweigert, Dirk Fuchs, Kenneth De Baets

**Affiliations:** 1 Paläontologisches Institut Und Museum, Universität Zürich, Karl-Schmid-Strasse 4, 8006 Zürich, Switzerland; 2 Staatliches Museum Für Naturkunde, Rosenstein 1, 70191 Stuttgart, Germany; 3 SNSB-Bayerische Staatssammlung Für Paläontologie Und Geologie, Richard-Wagner-Straße. 10, 80333 Munich, Germany; 4 GeoZentrum Nordbayern, Fachgruppe PaläoUmwelt, Friedrich-Alexander-University Erlangen-Nürnberg, Loewenichstr. 28, 91054 Erlangen, Germany

**Keywords:** Cephalopoda, Coleoidea, Jurassic, Palaeoecology, Taphonomy

## Abstract

Exceptional fossil preservation is required to conserve soft-bodied fossils and even more so to conserve their behaviour. Here, we describe a fossil of a co-occurrence of representatives of two different octobrachian coleoid species. The fossils are from the Toarcian Posidonienschiefer of Ohmden near Holzmaden, Germany. The two animals died in the act of predation, i.e. one had caught the other and had begun to nibble on it, when they possibly sank into hypoxic waters and suffocated (distraction sinking). This supports the idea that primitive vampyromorphs pursued diverse feeding strategies and were not yet adapted to being opportunistic feeders in oxygen minimum zones like their modern relative *Vampyroteuthis*.

## Introduction

Behavioural patterns of extinct animals can be studied either by trace fossils or ‘frozen behaviour’ (Boucot 1990; Radwański et al. 2009; Jenny et al. 2019; Hoffmann et al. 2020), i.e. when an animal became embedded in the sediment in a posture enshrining an aspect of its behaviour. Such cases are of special interest, because they may document interactions between individuals of one or more species (e.g., Radwański et al. 2009; Jenny et al. 2019; Hoffmann et al. 2020; Mapes et al. 2019). In the case of fossilized predatory behaviour, these fossils help reconstructing ancient food webs (e.g., Cohen et al. 1993; Dunne et al. 2008; Frey and Tischlinger 2012; Chevrinais et al. 2017; Jenny et al. 2019; Hoffmann et al. 2020; Hart et al. 2020). As recently shown, it can be a behavioural pattern that sometimes leads to ‘frozen behaviour’: Mapes et al. (2019) introduced the term ‘distraction sinking’ for cases, where aquatic organisms focused on certain activities such as eating, mating or else and did not control their position in the water column. In regions with poorly oxygenated water in greater depths, this distraction sinking caused the demise of these organisms (e.g., Mapes et al. 2019; Jenny et al. 2019; Hart et al. 2020).

Here, we present a slab with two fossil coleoid specimens in close association that was extracted from the Posidonienschiefer Formation (Posidonia Shale) of the famous conservation deposit of Holzmaden in Germany (‘Konservat-Lagerstätte’ sensu Seilacher 1970). The German Posidonienschiefer and its Central European equivalents in, e.g., France, Luxembourg, and Switzerland are well known for complete vertebrate skeletons as well as soft tissue-preservation of vertebrates and invertebrates (Hauff and Hauff 1981; Riegraf et al. 1984; Godefroit 1994; Röhl et al. 2001, 2002; Bottjer et al. 2002; Etter and Tang 2002). The most widely accepted explanation for the exceptional fossil preservation is the fluctuating oxygen content of bottom waters, which often were quite low (Röhl et al. 2001, 2002).

As far as exceptionally preserved coleoids are concerned, the Posidonienschiefer shares its importance with other Fossillagerstaetten such as, e.g., the Carboniferous of Bear Gulch (Landman and Davis 1988; Mapes et al. 2010, 2019; Klug et al. 2019), the Middle Jurassic of Christian Malford and Rixon Gate (Doyle and Shakides 2004; Wilby et al. 2004; Donovan 2006; Hart et al. 2016, 2020), the Middle Jurassic of La Voulte-sûr-Rhône (Fischer and Riou 1982a, b; Charbonnier 2009; Kruta et al. 2016), the Late Jurassic of Eichstätt, Nusplingen, Painten and Solnhofen (Fuchs 2006a, 2015; Klug et al. 2005, 2010, 2015,2016; Keupp et al. 2010), and the Late Cretaceous of Hâkel and Hâdjoula (Fuchs 2006b; Fuchs and Larson 2011a, b; Fuchs et al. 2009; Jattiot et al. 2015; Klug et al. 2021). As pointed out by Clements et al. (2016), the preservation and non-preservation of soft-tissues is linked with the original physiology of the coleoids. The material described here comprises two octobrachian coleoids, which both preserve extensive remains of phosphatised soft parts. There is a considerable literature explaining soft-tissue phosphatisation, which is not revised here in detail (Allison 1988; Briggs and Wilby 1996; Clements et al. 2016).

The Coleoidea evolved in the Late Devonian or Early Carboniferous and the split into Decabrachia (= ’Decapodiformes’) and Octobrachia (= Octopodiformes) happened likely during the Late Palaeozoic (e.g., Doyle et al. 1994; Voight 1997; Young et al. 1998; Haas 2002; Bizikov 2004; Lindgren et al. 2004; Fuchs 2006a; Strugnell et al. 2006; Kröger et al. 2011; Klug et al. 2016a, b, 2019). This subdivision and the names were introduced by Haeckel (1866: p. CXVI). For details, see Hoffmann (2015). In the Mesozoic, the decabrachians are mainly represented by proostracum-bearing belemnitids and diplobelids (Naef 1922; Kröger et al. 2011; Fuchs et al. 2013a, b; Klug et al. 2016a, b). At that time, octobrachians comprised gladius-bearing forms that superficially resemble modern day squids. While belemnites produced locally highly abundant fossils in the form of their calcified rostra, the fossil record of Mesozoic octobrachians is more sparse and limited to strata with exceptional fossil preservation of weakly or non-mineralized body parts such as the conservation deposits listed above. In this latter case, however, octobrachians with soft-tissue preservation can be quite abundant and their anatomy has become well-known (e.g., Naef 1922; Fuchs 2006a, 2015; Klug et al. 2005, 2010, 2015; Keupp et al. 2010; Hart et al. 2020; Fuchs et al. 2016; Donovan and Fuchs 2016; Kruta et al. 2016).

In the fossil described here, one smaller octobrachian rests in the arm crown of a much larger octobrachian of a different taxon. Although octobrachians can be found in the Posidonienschiefer occasionally, they are not that abundant that such a joint occurrence by chance would be very likely. Hence, we decided to (1) describe this fossil in detail, (2) discuss the palaeobiological implications, and (3) put it into the palaeoecological and (4) taphonomical context.

## Material

The main specimen was found and skilfully prepared by the amateur collector Dieter Weber (Rechberghausen), purchased from the latter by one of us (G.S.) and subsequently donated to the Staatliches Museum für Naturkunde Stuttgart (acronym SMNS). It originates from the Early Jurassic (Toarcian) Posidonienschiefer Formation of a now abandoned quarry opposite to the present golf club area at Ohmden, c. 2.5 kms northeast of Holzmaden. The exact finding level of the specimen is the c. 60 cm thick ʻUnterer Schieferʼ bed, a bituminous claystone, which is Early Toarcian, uppermost Semicelatum to lowermost Falciferum (= Serpentinum) Zone in age (Riegraf et al. 1984). Within the entire Posidonienschiefer Formation, this interval shows the highest kerogen content and is very rich in pyrite. Additionally, the claystone is finely laminated and lacks bioturbation, thus suggesting hostile environmental conditions at and within the sea floor as a regional expression of the global Early Toarcian Anoxic Event (e.g., Jenkyns and Clayton 1986; Jenkyns 1988) resulting in the above mentioned preservation conditions. The ʻUnterer Schieferʼ is world-renowned for containing various articulated vertebrates (e.g., ichthyosaurs, marine crocodiles, fishes) and nektic invertebrates (ammonoids, belemnites and other coleoids), whereas benthic animals are quite rare (see list in Riegraf et al. 1984).

## Results

**Fig. 1:**
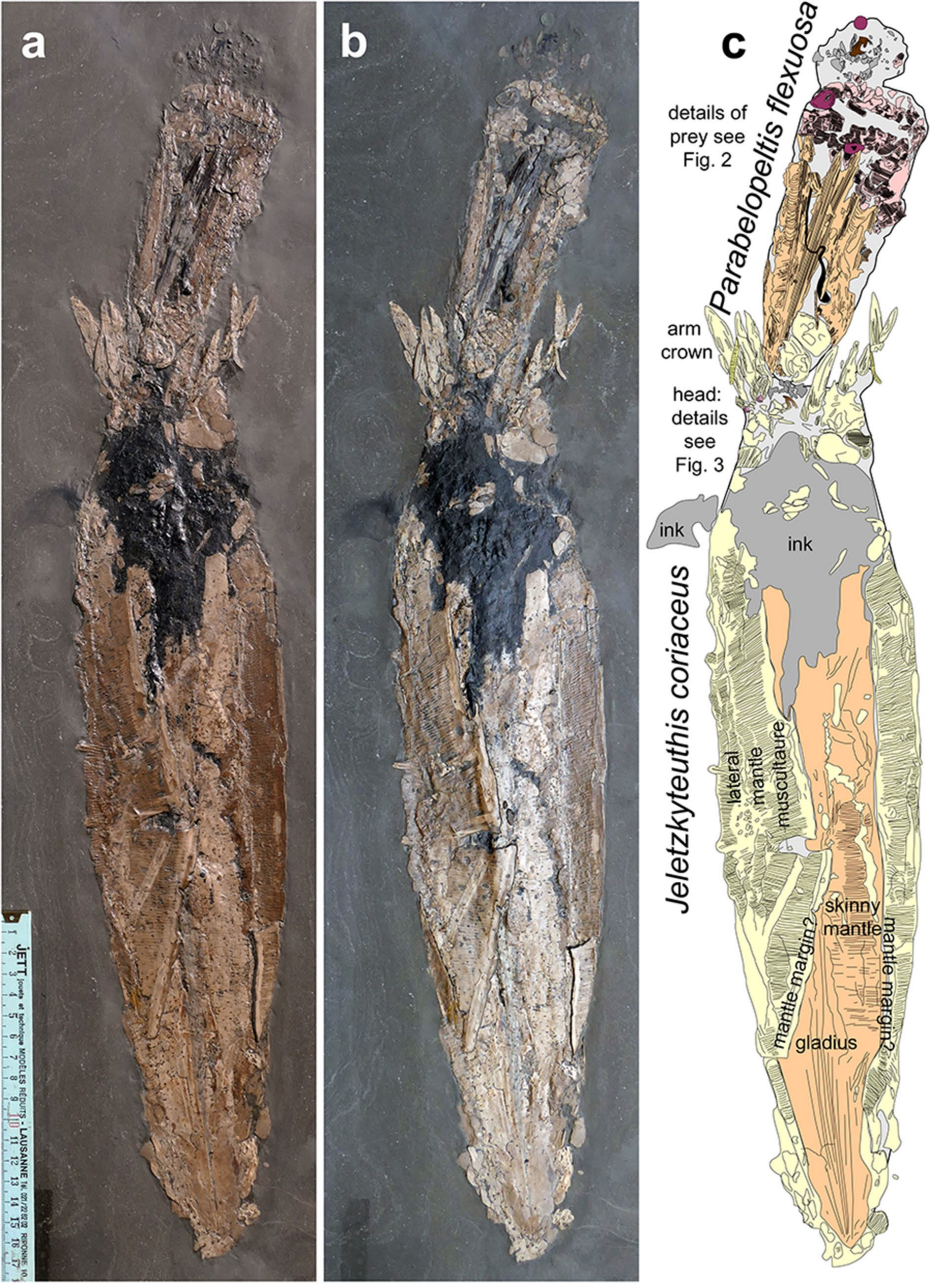
Taphocoenosis of *Jeletzkyteuthis coriacea* with a smaller specimen of *Parabelopeltis flexuosa*, SMNS 70,623, Early Toarcian, Semicelatum to lowermost Serpentinum Zone, Ohmden, Germany. **a**, **b** photos taken with different lights. Note how morphological details appear differently in the two images. **c** Camera lucida drawing after **b**

The two more or less complete octobrachian fossils lie on a large slab of Posidonienschiefer, which measures 640 × 170 mm (Fig. 1). The larger of the two coleoids is 465 mm long including arms. The smaller specimen is 167 mm long, i.e. it measures less than 40% of the preserved body length of the larger animal. The smaller specimen is assigned to *Parabelopeltis flexuosa* (Münster 1843) based on characteristic growth increments visible on the posterior left side of the gladius (Fuchs and Weis 2008: fig. 3).

The larger specimen is more difficult to determine because the muscular mantle covers much of the gladius, thus obscuring gladius details. The body proportions are conspicuously long and slender. Among the common octobrachians in the Posidonienschiefer, only the slender gladiuses of *Paraplesioteuthis sagittata* (Münster 1843) (Prototeuthidina) and *Jeletzkyteuthis coriacea* (Quenstedt, 1849) (Loligosepiina) share this torpedo-shaped mantle outline. The remarkable size of the specimen points to *Jeletzkyteuthis coriacea*, whose gladiuses regularly exceed 200 mm in length (Guerin-Franiatte and Gouspy 1993; Fuchs and Weis 2008). Gladiuses of *Paraplesioteuthis sagittata,* by contrast, rarely reach 200 mm. Systematics and morphological terminologies used below follow Fuchs (2020).

### *Parabelopeltis flexuosa* (Figs. 1, 2)

**Fig. 2:**
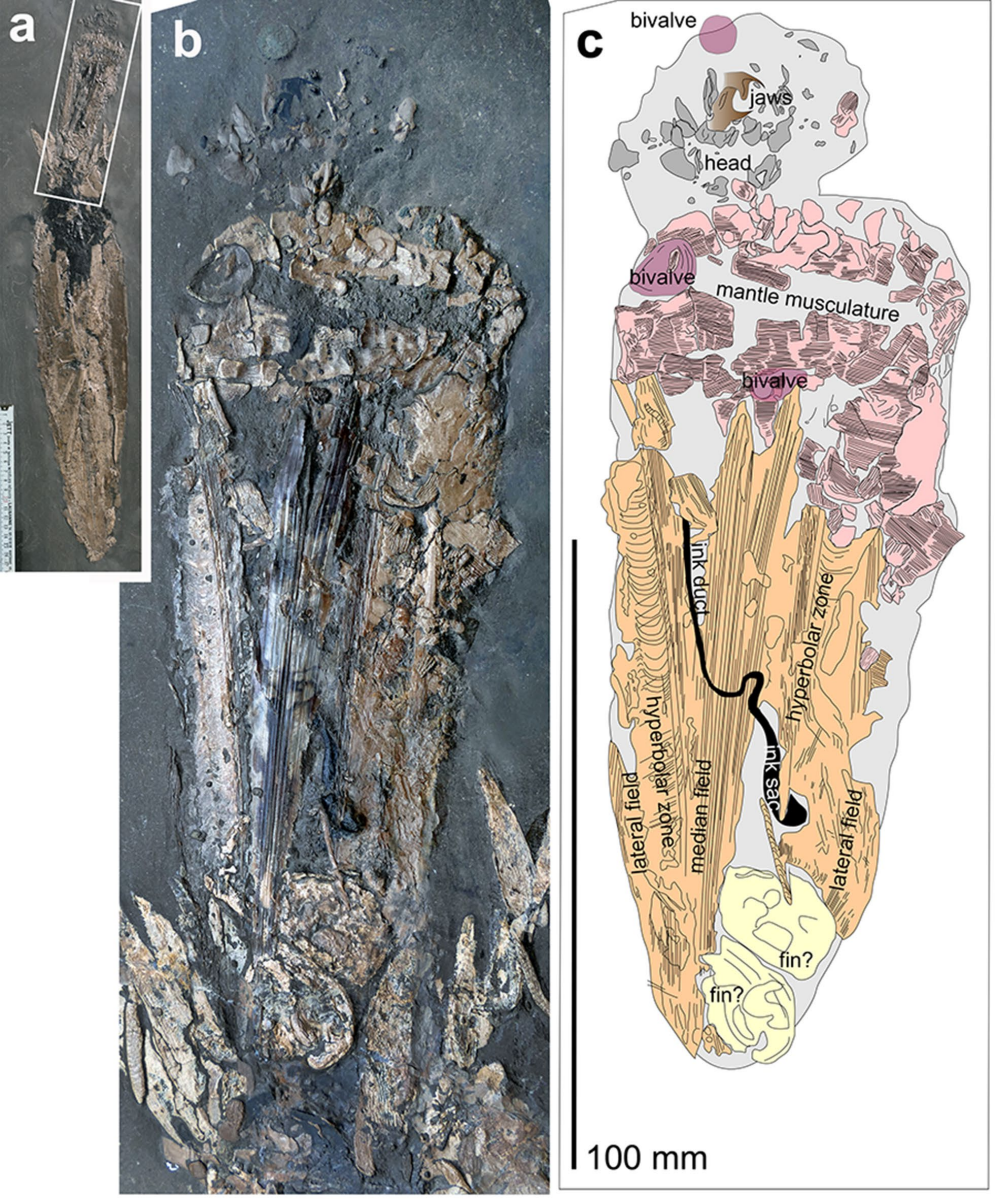
Detail of Fig. 1 showing only the prey-coleoid *Parabelopeltis flexuosa*; SMNS 70,623, Early Toarcian, Semicelatum to lowermost Serpentinum Zone, Ohmden, Germany. **a** Overview of the fossil with a rectangle showing the localisation of **b**. **b** Detail of Fig. 1b showing the prey coleoid and the arm crown of the predator. **c** Drawing after **b** to show the anatomical details of the coleoid

The smaller octobrachian is 167 mm long and maximally 57 mm wide. Of the 167 mm, 138 mm are occupied by the mantle and gladius (mantle length). The 29 mm long head region comprises some dark grey phosphatic shards of various sizes, which cannot be assigned to distinct body parts unequivocally, except for the jaws. Remarkably, both upper and lower jaws are preserved; they display the elongate and pointed rostra as well as the outer lamella, but posteriorly, preservation of both jaws deteriorates, probably because of decreasing sclerotisation. In Fig. 2c, the jaws are drawn in brown. In the fossil, they have a very dark grey colour. In dorsoventral direction, the jaw visible above the other is about 5 mm high, while the other jaw measures about 7 mm.

Gladius preservation suggests that the specimen is seen in dorsal aspect, which explains its only partial muscular cover. The transversely striated mantle musculature is predominantly visible in the anterior part of the mantle and on the right flank. Additionally, ink sac and ink duct extend over about 50 mm; they are partially covered by the gladius and partially still visible through the gladius. At the posterior end of the gladius, two phosphatic oval structures are preserved, which measure about 20 × 14 mm. These are tentatively interpreted as fins, although finer structures helping to test this interpretation are missing. This interpretation is mainly based on the position, the dimensions and the presence of two on one side (presumably four in total) as in the juveniles of the modern *Vampyroteuthis infernalis* (e.g., Hoving and Robison 2012) or in adult *Trachyteuthis hastiformis* (Donovan et al. 2003; Fuchs 2006a, 2015) and *Plesioteuthis prisca* (Klug et al. 2015).

The gladius is partially covered by mantle and is probably about 135 mm long (if measured from the apex to the anterior mantle edge) although it is exposed only over 110 mm. Due to compaction, it is strongly flattened, but the main structures such as median and lateral fields as well the hyperbolar zones are recognizable. Circa 25 mm of the longitudinally ribbed median field is visible. It carries about 30 longitudinal ribs. The adjacent left hyperbolar zone is 2 to 3 mm wide and smoothly arcuated. The lateral field is c. 5 mm wide and begins where growth increments turn backwards. The gladius margin lies close to it with a distance of a few millimetres. The conus is not well discernible.

### Jeletzkyteuthis coriacea

The second coleoid is much larger and preserves more detail of the head, particularly of the arm crown, but the gladius is largely covered by musculature, hampering unequivocal taxon identification. The entire individual measures 465 mm in length including the head and arm crown as it is preserved. The mantle length is about 400 mm with a width of up to about 100 mm. Gladius length likely corresponds to mantle length.

The arm crown appears to be complete. It displays eight elongate and pointed phosphatized structures (four on both sides of the midline), which measure between 40 and 50 mm in length and between 5 and 9 mm in width. They exhibit a longitudinal striation that likely represents phosphatized longitudinal muscle fibres (cf. Fuchs 2006a, 2015; Donovan and Fuchs 2016). The arms embrace the posterior end of the body of the smaller coleoid in a nearly symmetrical fashion with four arms on each side of the conus of the supposed prey.

Additionally, two worm-shaped structures are arranged more or less symmetrically. They are 16 and 22 mm long, the longer having a diameter of 3 mm and the other being 2 mm wide. In contrast to the arms, these structures are transversely striated with about 14 to 20 such striae per 10 mm length. Because of their symmetric arrangement, their transverse striation, and the presence of only two of these structures between the arms, we suggest that these structures are homologous to the long filaments of *Vampyroteuthis infernalis* (Hoving and Robison 2012).

**Fig. 3:**
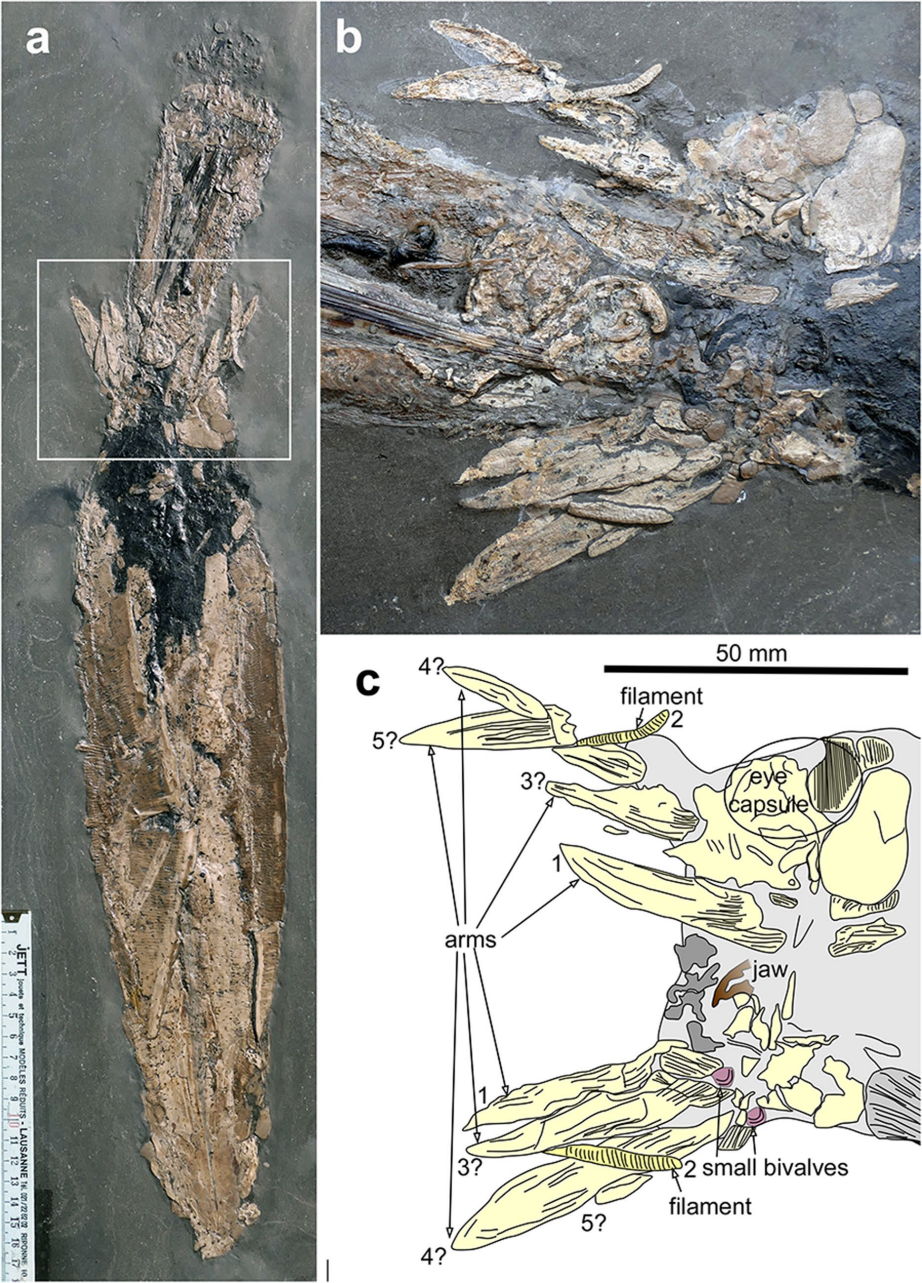
Details of the head and arm crown of the predator (*Jeletzkyteuthis coriacea*), SMNS 70623, Early Toarcian, Semicelatum to lowermost Serpentinum Zone, Ohmden, Germany. **a** Overview of the fossil with a rectangle showing the localisation of the details shown in **b**, **c**. **b** Detail of showing head and arm crown still surrounding the posterior end of the prey coleoid

In contrast to the other coleoid, the jaw is much less well preserved. The 9 mm long curved rostrum of one of the jaws (probably the upper jaw) is visible. The lower jaw and the rest of the partially exposed upper jaw are covered by soft tissues. Remarkably, much of the head is preserved as a dark grey mass devoid of finer structure. Only the arms and the filaments as well as some irregular parts on both sides of the head are preserved in beige-coloured phosphate. In Fig. 3b, c, there is a kidney-shaped structure, which is 23 mm long and 11 mm wide. Because of its position and size, we think that this is an eye capsule. Behind this, there is a pear-shaped structure of almost the same dimensions and proportions (23 × 14 mm). Assuming that the plain of bilateral symmetry of the animal corresponds approximately to the plain of symmetry of the fossil, it appears unlikely that this is the other eye. Instead, this might be the optic lobe of the cephalic cartilage (e.g., Chung et al. 2020).

The mantle musculature is quite completely preserved, displaying the characteristic transverse striation. There are about 9 to 12 transverse striae per 10 mm in the mantle musculature. The mantle musculature is arranged in two long flaps that extend from both sides over the dorsal gladius surface. These two mantle flaps are up to about 30 mm wide on the right and up to about 50 mm wide on the left side. On both sides, the transverse striation ends in a longitudinal band that is about 5 to 7 mm wide. These bands might represent the attachment ligaments of the mantle musculature, which connected it to the gladius (cf. Fuchs et al. 2015). The right flap appears to be still in contact with the gladius margin, whereas the left side seemingly detached and covered parts of the gladius. The gladius is therefore only partially exposed over a length of about 332 mm, i.e. the anterior 68 mm are covered by a dark substance that might be ink-stained soft tissues and phosphatized mantle musculature (cf. Klug et al. 2021). Posteriorly, the acute conus part is exposed. A diverging structure that opens at an angle of less than 10° may be interpreted as a very slender median field (Fuchs 2020).

## Discussion

### Arm crown of Early Jurassic vampyromorphs

The gladii of many Jurassic octobrachians superficially resemble the homologous hard parts of decabrachians such as, e.g. the pens of living *Loligo* and *Ommastrephes* (similar to, e.g., *Teudopsis* and *Plesioteuthis*, respectively) or the cuttlebone of *Sepia* (similar to, e.g. *Trachyteuthis*). This similarity led to discussions about the systematic position of Mesozoic gladius-bearing coleoids (“fossil teuthids” after Naef 1922 and Jeletzky 1966; see also, e.g., Bandel and Leich 1986; Doyle et al. 1994; Fuchs and Iba 2015; Fuchs 2016). Nevertheless, the number of known synapomorphies (mainly soft tissue characters) of modern *Vampyroteuthis,* modern octopods, and Mesozoic gladius-bearing coleoids has been increasing in the past decades, supporting the hypothesis of octobrachian affinities of Prototeuthidina, Loligosepiina, and Teudopseina (e.g., Donovan et al. 2003; Fuchs et al. 2013a, b, 2015; Donovan and Fuchs 2016).

One of the strongest arguments is the number of preserved arms. In each of the latter groups, eight muscular arms have been counted. However, loligosepiids, which are commonly seen as vampyromorph stem representatives (e.g., Engeser 1988; Doyle et al. 1994; Fuchs 2020), should exhibit a fifth arm pair, because *Vampyroteuthis infernalis* is indeed typified by an additional filamentous arm pair in dorsolateral position (e.g., Young et al. 1998).

The larger of the two loligosepiids described here preserves remains of four rather symmetrically arranged arm pairs. Between the arms, two narrow and elongate, transversely striated structures are preserved, which are much thinner than the muscular arms and differently preserved. This is not the only material preserving these filaments. A loligosepiid specimen that is on display in the Urweltmuseum Hauff in Holzmaden, Germany, also shows remains of what we consider the filaments and thus, this is not an artefact (https://commons.wikimedia.org/wiki/File:Loligosepia.JPG accessed on January 11^th^ 2021).

There are several possible interpretations of the structure: These could be a hectocotylised arm pair (i), tentacles as known from modern sepiids, oegopsids and other decabrachians (ii), or filaments as known from the Recent *Vampyroteuthis* (iii). The male hectocotylus (i) is used to transfer spermatophores to the female. Hectocotylisation (Müller 1853; Robson 1929; Palacio 1973; Jereb et al. 2014) may include a single arm (e.g., *Argonauta*, *Octopus*, *Tremoctopus*) or an arm pair (e.g., *Abraliopsis*, *Octopus*). It is often longer than the other arms and always modified; its distal end usually lacks suckers, is somewhat wider and with a distinct surface structure. In *Jeletzkyteuthis*, this pair of thinner appendages does differ from the other four arm pairs, but it is much thinner and lacks the distal modifications (we cannot rule out that it is simply not preserved). Hence, we consider the hypothesis that these are hectocotyli as unlikely. Since there is no trace preserved of a structure resembling the club at the end of decabrachian tentacles (as, e.g. in the giant squid *Architeuthis* or the colossal squid *Mesonychoteuthis*; Compagno Roeleveld and Lipinski 1991; Lordan et al. 1998; Rosa et al. 2017), these are probably not tentacles as known from modern decabrachians (ii). Instead, we suggest that these structures are homologues of the filaments of *Vampyroteuthis* (iii) because of their position in the arm crown (supposedly between arm pairs 1 and 3, which is not testable in this specimen), their specific quality such as their thickness (much thinner than the arms) and the transverse striation possibly due to contraction (cf. Hoving and Robison 2012).

### Predatory behaviour of Jurassic octobrachians

Cephalopods are important predators in marine ecosystems (Villaneuva et al. 2017). One of the most iconic exceptions is the modern vampire squid (*Vampyroteuthis infernalis*), which has developed a unique ecology adapted to being slow opportunistic feeders in the oxygen minimum zone using their retractable filaments (Hoving and Robison 2012; Golikov et al. 2019). However, it is still unresolved when this feeding strategy first appeared. There is independent support that earlier vampire squids inhabited epicontinental seas, while the earliest record of species known from bathyal habitats with support for bottom-water anoxia only appeared in the Oligocene (Košťák et al. 2021).

A mutual predator–prey-relationship between coleoid cephalopods and vertebrates dates back deep in time (Landman and Davis 1988; Přikryl et al. 2012; Klug et al. 2019). Fish eat cephalopods and vice versa (Hess and Toll 1981; Nixon 1985, 1987, 1988; Boucot 1990; Přikryl et al. 2012; Jenny et al. 2019; Hart et al. 2020). Although *Vampyroteuthis infernalis* has usually been observed passively floating to collect detritus including small planktic organisms with their arm filaments, occasionally also remains of larger, fast moving prey including crustaceans, squid and fish have been recovered from stomach contents (Seibel in Golikov et al. 2019). Predation of coleoids on other coleoids is not rare in recent cephalopods (e.g., *Loligo*: Coelho et al. 1997; *Architeuthis*: Lordan et al. 1998) and even cannibalistic behavior was documented in modern (Boletzky and Hanlon 1983; Nixon 1985; Breiby and Jobling 1985; Hanlon and Forsythe 2008) and extinct coleoids (*Belemnotheutis antiqua*: Wilby et al. 2004; *Idahoteuthis parisiana*: Doguzhaeva et al. 2018).

Fossilised evidence for predation by coleoids on coleoids is rare. Some of the best examples were documented from the Oxford Clay in Wiltshire, UK. In these cases, the octobrachian *Mastigophora* caught the smaller decabrachian belemnoid *Belemnotheutis* and *Belemnotheutis* showed cannibalistic behavior between individuals of different sizes (Wilby et al. 2004).

In the fossil portrayed here, two extinct octobrachians were embedded and fossilized in an eternal embrace. This situation could be explained by (i) chance (taphonomy), (ii) reproductive behavior (even if between different species), or (iii) a predation attempt. We consider a purely taphonomic explanation of this taphocoenosis (i) as unlikely because complete coleoid fossils are not very common (see also Wilby et al. 2004: p. 1176). As discussed by, e.g. Jenny et al. (2019) and Hart et al. (2020) for cases of predation of coleoids on actinopterygian fishes, a parallel orientation would be more likely in the case of, e.g., current alignment. In these cases, the interpretation of frozen behavior sensu Boucot (1990) is supported by the fact that several such fossils have been found where coleoids hold fishes in more or less the same way, in some cases even with skeletal fractures visible in the prey (Jenny et al. 2019; Hart et al. 2020).

As far as reproductive behavior is concerned (ii), we cannot rule out that this represents an erroneous attempt between different species. Nevertheless, the fact that these are different species and the way the larger individual holds the smaller one does not correspond well with the mating behavior of living cephalopods. In modern coleoids, the male usually faces the head of the female either from the front or from the side (e.g., Corner and Moore 1980; Boyle 1987; Boal 1997, 2006; Hanlon et al. 2005; Ylitalo et al. 2019). Hence, we consider this hypothesis to be unlikely as well.

**Fig. 4:**
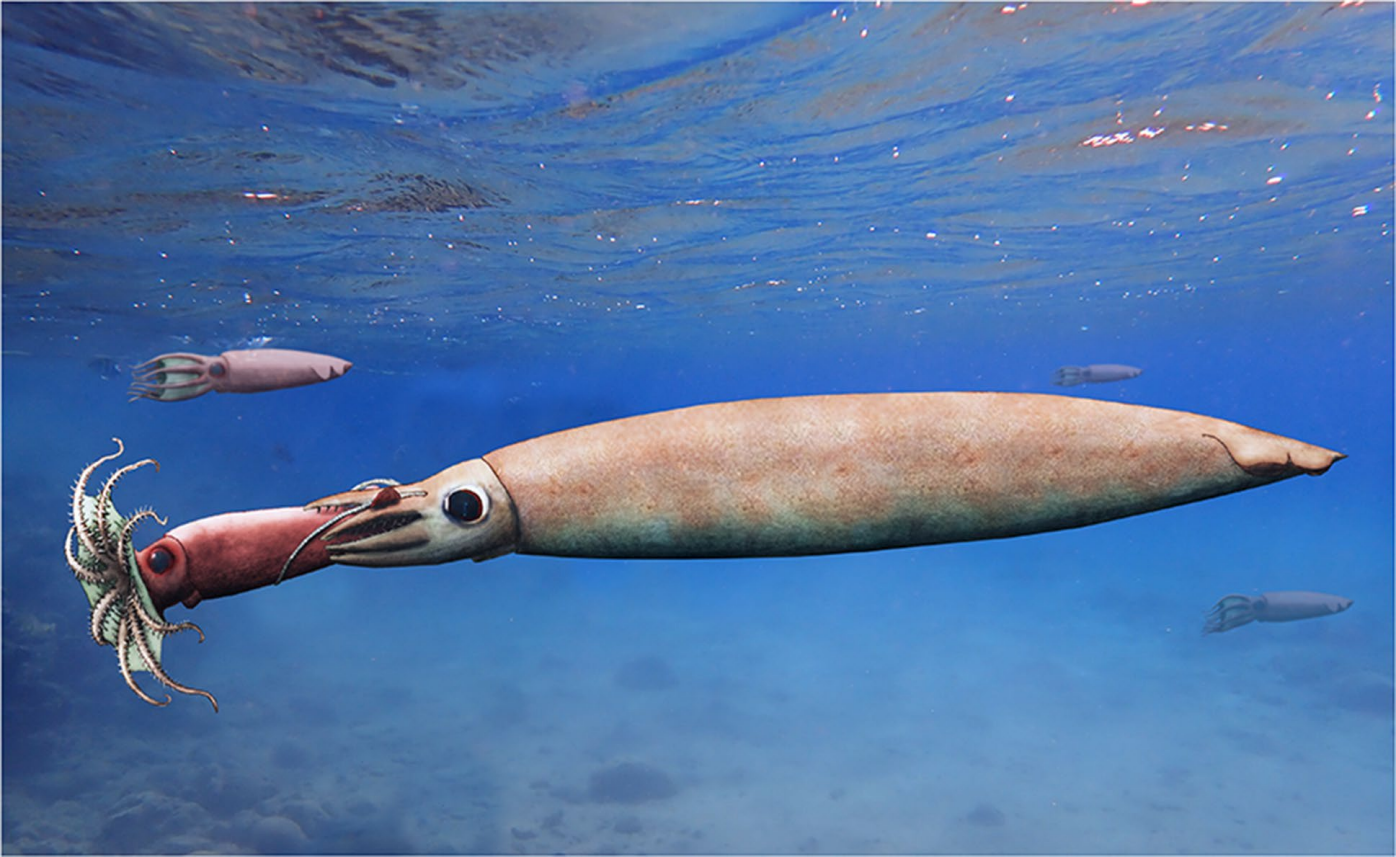
Reconstruction of the Toarcian *Jeletzkyteuthis coriacea* catching a smaller specimen of *Parabelopeltis flexuosa*

Coleoid cephalopods are known to be predators from hatching onward (e.g., Nixon 1985, 1987) with some rare exceptions (Fernández-Álvarez et al. 2018). Additionally, fossilized cases of incomplete (prey not completely ingested) and completed predation (crop, stomach or gut contents) of cephalopods (iii) have been documented by, e.g., Landman and Davis (1988), Mapes et al. (2010), Doguzhaeva et al. (2018), Jenny et al. (2019), Klug et al. (2019), and Hart et al. (2020). Most of these authors reported on predation on vertebrates, while Doguzhaeva et al. (2018) demonstrated that *Idahoteuthis* fed on another coleoid and Wilby et al. (2004) showed the same for *Belemnotheutis*. The fossil presented here shows two loligosepiid vampyromorphs, where the larger individual embraces the posterior end of the smaller individual. Taking into account that the smaller individual is held by the larger and that they belong to different species, we consider that the larger animal caught the smaller animal in order to feed on it (Fig. 4).

The question arises as to why the coleoid prey was not further processed. The two animals are embedded in a dark claystone, which was deposited during the Toarcian in the Germanic Basin. As shown by Röhl et al. (2001, 2002), anoxic and hypoxic conditions occurred in the waters near the sediment surface repeatedly in that time and region, explaining the scarcity of scavenging and the abundance of exceptional fossil preservation (e.g., Allison 1988; Briggs and Wilby 1996; Clements et al. 2016). As suggested for other cases of fossilized predation by coleoids, we assume that the successful predator did not focus on the water depth when it began to eat and started sinking (e.g., Jenny et al. 2019; Mapes et al. 2019). Eventually, the animals reached the lower part of the water body with insufficient oxygen levels causing their asphyxiation. Accordingly, this would be another case of ‘distraction sinking’ (Mapes et al. 2019). This would also be in line with the interpretation that early vampire squid were living in epicontinental seas and not yet adapted to low oxygen habitats (Košťák et al. 2021). The only question, which remains to be answered is whether the Early Jurassic forms described here were already using their filaments for detritus feeding on the side.

## Conclusions

We document two fossilized octobrachian coleoids from the Early Jurassic Posidonienschiefer of southern Germany. Both specimens are more or less completely preserved including most of their soft parts. The arm crown of the larger specimen, *Jeletzkyteuthis coriacea* (Vampyromorpha), counts eight muscular arms plus a pair of worm-shaped structures that we interpret as a fifth dorsolateral arm pair homologous to the filaments of extant *Vampyroteuthis*. If this interpretation is correct, the long filaments of extant *Vampyroteuthis* arose from a shorter thread-like arm pair.

The larger individual holds the posterior end of the smaller in its arms, with the apex very close to its jaws. We suggest that this is a case of frozen predatory behaviour of two different octobrachian species. Their preservation is explained by distraction sinking. This finding is a new building block helping to reconstruct the food web of the Early Jurassic of the Germanic Basin.

## Data Availability

The single specimen illustrated and described is stored at the Staatliches Museum für Naturkunde in Stuttgart, Germany.

## References

[CR1] Allison PA (1988). Phosphatized soft-bodied squids from the Jurassic Oxford Clay. Lethaia.

[CR2] Bandel K, Leich H (1986). Jurassic Vampyromorpha (dibranchiate cephalopods). Neues Jahrbuch für Geologie und Paläontologie, Monatshefte.

[CR3] Bizikov VA (2004). The shell in Vampyropoda (Cephalopoda): Morphology, functional role and evolution. Ruthenica Supplement.

[CR4] Boal JG (1997). Female choice of males in cuttlefish (Mollusca: Cephalopoda). Behaviour.

[CR5] Boal JG (2006). Social recognition: A top down view of cephalopod behaviour. Vie et milieu.

[CR6] Boletzky S, Hanlon RT (1983). A review of the laboratory maintenance, rearing and culture of cephalopod molluscs. Memoirs of Museum Victoria.

[CR7] Bottjer DJ, Etter W, Hagadorn JW, Tang CM (2002). Fossil-Lagerstätten: Jewels of the Fossil Record.

[CR8] Boucot, A. J. (1990). *Evolutionary paleobiology of behavior and coevolution*. Elsevier; Amsterdam. p. 1–725.

[CR9] Boyle, P. R. (1987). *Cephalopod Life Cycles. Vol. 2: Comparative Reviews.* Academic Press, London.

[CR10] Breiby A, Jobling M (1985). Predatory role of the flying squid (*Todarodes sagittatus*) in North Norwegian waters. NAFO Scientific Council Studies.

[CR11] Briggs DEG, Wilby PR (1996). The role of the calcium carbonate-calcium phosphate switch in the mineralization of soft-bodied fossils. Journal of the Geological Society, London.

[CR12] Charbonnier S (2009). Le Lagerstätte de La Voulte: un environnement bathyal au Jurassique. Mémoires du Muséum national d'Histoire naturelle.

[CR13] Chevrinais M, Jacquet C, Cloutier R (2017). Early establishment of vertebrate trophic interactions: Food web structure in Middle to Late Devonian fish assemblages with exceptional fossilization. Bulletin of Geosciences.

[CR14] Chung W-S, Kurniawan ND, Marshall NJ (2020). Toward an MRI-based mesoscale connectome of the squid brain. Science.

[CR18] Clements T, Colleary C, De Baets K, Vinther J (2016). Buoyancy mechanisms limit preservation of coleoid cephalopod soft tissues in Mesozoic lagerstätten. Palaeontology.

[CR19] Coelho M, Domingues P, Balguerias E, Fernandez M, Andrade JP (1997). A comparative study of the diet of *Loligo vulgaris* (Lamarck, 1799) (Mollusca:Cephalopoda) from the south coast of Portugal and the Saharan Bank (Central-East Atlantic). Fisheries Research.

[CR20] Cohen JE, Pimm SL, Yodzis P, Saldaña J (1993). Body sizes of animal predators and animal prey in food webs. Journal of Animal Ecology.

[CR01] Compagno Roeleveld MA, Lipinski MR (1991). The giant squid *Architeuthis* in southern African waters. Journal of Zoology.

[CR22] Corner BD, Moore HT (1980). Field observations on the reproductive behavior of Sepia latimanus. Micronesica.

[CR23] Doguzhaeva LA, Brayard A, Goudemand N, Krumenacker LJ, Jenks JF, Bylund KG, Fara E, Olivier N, Vennin E, Escarguel G (2018). An Early Triassic gladius associated with soft tissue remains from Idaho, USA- a squid-like coleoid cephalopod at the onset of Mesozoic Era. Acta Palaeontologica Polonica.

[CR25] Donovan DT (2006). Phragmoteuthida (Cephalopoda: Coleoidea) from the Lower Jurassic of Dorset, England. Palaeontology.

[CR26] Donovan DT, Doguzhaeva LA, Mutvei H (2003). Two pairs of fins in the Late Jurassic coleoid *Trachyteuthis* from southern Germany. Berliner Paläobiologische Abhandlungen.

[CR27] Donovan DT, Fuchs D (2016). Part M, chapter 13: fossilized soft tissues in Coleoidea. Treatise Online.

[CR28] Doyle P, Donovan DT, Nixon M (1994). Phylogeny and systematics of the Coleoida. Paleontological Contributions, University of Kansas.

[CR29] Doyle P, Shakides EV (2004). The Jurassic belemnite suborder Belemnotheutina. Palaeontology.

[CR30] Dunne J, Williams RJ, Martinez ND, Wood RA, Erwin DH (2008). Compilation and network analyses of Cambrian food webs. PLoS Biology.

[CR31] Engeser, T. (1988). Vampyromorpha ("Fossile Teuthiden"). In: F. Westphal (Ed.), *Fossilium Catalogus. I: Animalia* (Vol. 130, pp. 1–167). Amsterdam, Kugler Publications.

[CR32] Etter, W. & Tang, C. M. (2002). Posidonia shale: Germany’s Jurassic marine park. In: Bottjer, D. J., Etter, W., Hagadorn, J. W. & Tang, C.: *Exceptional Fossil Preservation*. Columbia University Press, New York, pp 265–291.

[CR33] Fernández-Álvarez FÁ, Machordom A, García-Jiménez R, Salinas-Zavala CA, Villanueva R (2018). Predatory flying squids are detritivores during their early planktonic life. Scientific Reports.

[CR34] Fischer JC, Riou B (1982). Les Teuthoïdes (Cephalopoda, Dibranchiata) du Callovien inférieur de La Voulte-sur-Rhône (Ardèche, France). Annales de Paléontologie.

[CR35] Fischer JC, Riou B (1982). *Vampyronassa rhodanica* nov. gen. nov sp., vampyromorphe (Cephalopoda, Coleoidea) du Callovien inférieur de la Voulte-sur-Rhône (Ardèche, France). Annales de Paléontologie.

[CR36] Frey E, Tischlinger H (2012). The Late Jurassic pterosaur *Rhamphorhynchus*, a frequent victim of the ganoid fish *Aspidorhynchus*?. PLoS ONE.

[CR37] Fuchs D (2006). Fossil erhaltungsfähige Merkmalskomplexe der Coleoidea (Cephalopoda) und ihre phylogenetische Bedeutung. Berliner Paläobiologische Abhandlungen.

[CR38] Fuchs D (2006). Morphology, taxonomy and diversity of vampyropod Coleoids (Cephalopoda) from the Upper Cretaceous of Lebanon. Memorie della Società Italiana di Scienze Naturali.

[CR39] Fuchs, D. (2015). Tintenfische (Coleoidea, Endocochleata, Dibranchiata). In: Arratia, G., Schultze, H.-P., Tischlinger, H. & Viohl, G. (eds.), *Solnhofen – Ein Fenster in die Jurazeit. Vol. 1+2* [in German]. 229–238. Pfeil, Munich.

[CR40] Fuchs, D. (2016). Part M, Coleoidea, Chapter 9B: The gladius and gladius vestige in fossil Coleoidea. *Treatise Online, 83*, 1–23.

[CR41] Fuchs, D. (2020). Part M, Coleoidea, Chapter 23G: Systematic Descriptions: Octobrachia. *Treatise Online, 138*, 1–52.

[CR42] Fuchs D, Bracchi G, Weiss R (2009). New Octopods (Cephalopoda: Coleoidea) from the Late Cretaceous (upper Cenomanian) of Hâkel and Hâdjoula, Lebanon. Palaeontology.

[CR43] Fuchs D, Iba Y, Ifrim C, Nishimura T, Kennedy J, Keupp H (2013). *Longibelus* gen. nov., a new Cretaceous coleoid genus linking Belemnoidea and Decabrachia. Palaeontology.

[CR44] Fuchs D, Iba Y (2015). The gladiuses in coleoid cephalopods: homology, parallelism, or convergence?. Swiss Journal of Palaeontology.

[CR45] Fuchs D, Iba Y, Tischlinger H, Keupp H, Klug C (2015). On the locomotion system of fossil Coleoidea (Cephalopoda) and its phylogenetic significance. Lethaia.

[CR46] Fuchs D, Keupp H, Schweigert G (2013). First record of a complete arm crown of the Early Jurassic coleoid *Loligosepia* (Cephalopoda). Paläontologische Zeitschrift.

[CR47] Fuchs D, Larson NL (2011). Diversity, morphology, and phylogeny of coleoid cephalopods from the Upper Cretaceous Plattenkalks of Lebanon - Part I: Prototeuthidina. Journal of Paleontology.

[CR48] Fuchs D, Larson NL (2011). Diversity, morphology and phylogeny of coleoid cephalopods from the Upper Cretaceous Plattenkalks of Lebanon - Part II: Teudopseina. Journal of Paleontology.

[CR49] Fuchs D, Weis R (2008). Taxonomy, morphology and phylogeny of Lower Jurassic loligosepiid coleoids (Cephalopoda). Neues Jahrbuch für Geologie und Paläontologie Abhandlungen.

[CR51] Godefroit P (1994). Les reptiles marins du Toarcien Jurassique inférieur belgo-luxembourgeois. Mémoire pour servir d'explication aux cartes géologiques et minières de la Belgique.

[CR52] Golikov AV, Ceia FR, Sabirov RM, Ablett JD, Gleadall IG, Gudmundsson G, Hoving HJ, Judkins H, Pálsson J, Reid AL, Rosas-Luis R, Shea EK, Schwarz R, Xavier JC (2019). The first global deep-sea stable isotope assessment reveals the unique trophic ecology of Vampire Squid *Vampyroteuthis infernalis* (Cephalopoda). Scientific reports.

[CR53] Guerin-Franiatte S, Gouspy C (1993). Decouverte des Cephalopodes Teuthides (Coleoidea) dans le Lias superieur de Haut-Marne, France. GEOBIOS, memoire speciale.

[CR54] Haas W (2002). The evolutionary history of the eight-armed Coleoidea. Abhandlungen der Geologischen Bundesanstalt.

[CR55] Haeckel E (1866). Generelle Morphologie der Organismen.

[CR56] Hanlon RT, Forsythe JW (2008). Sexual cannibalism by *Octopus cyanea* on a Pacific coral reef. Marine and Freshwater Behaviour and Physiology.

[CR57] Hanlon RT, Naud M-J, Shaw PW, Havenhand JN (2005). Transient sexual mimicry leads to fertilization. Nature.

[CR58] Hart MB, de Jonghe A, Page KN, Price GD, Smart CS (2016). Exceptional accumulations of statoliths in association with the Christian Malford lagerstätte (Callovian, Jurassic) in Wiltshire, United Kingdom. Palaios.

[CR59] Hart MB, Arratia G, Moore C, Ciotti BJ (2020). Life and death in the Jurassic seas of Dorset, Southern England. Proceedings of the Geologists’ Association.

[CR60] Hauff B, Hauff RB (1981). Das Holzmadenbuch.

[CR61] Hess SC, Toll RB (1981). Methodology for specific diagnosis of cephalopod remains in stomach contents of predators with reference to the broadbill swordfish *Xiphias gladius*. Journal of Shellfish Research.

[CR62] Hoffmann R (2015). The correct taxon name, authorship, and publication date of extant ten-armed coleoids. Paleontological Contributions.

[CR63] Hoffmann R, Stevens K, Keupp H, Simonsen S, Schweigert G (2020). Regurgitalites – a window into fossil food webs. Journal of the Geological Society.

[CR64] Hoving H-JT, Robison BH (2012). Vampire squid: Detritivores in the oxygen minimum zone. Proceedings of the Royal Society B: Biological Sciences.

[CR65] Jattiot R, Brayard A, Fara E, Charbonnier S (2015). Gladius-bearing coleoids from the Upper Cretaceous Lebanese Lagerstätten: diversity, morphology, and phylogenetic implications. Journal of Paleontology.

[CR66] Jeletzky JA (1966). Comparative morphology, phylogeny and classification of fossil Coleoidea. Paleontological contributions, University of Kansas Mollusca.

[CR67] Jenkyns HC (1988). The early Toarcian (Jurassic) anoxic event: Stratigraphic, sedimentary, and geochemical evidence. American Journal of Science.

[CR68] Jenkyns HC, Clayton CJ (1986). Black shales and carbon isotopes in pelagic sediments from the Tethyan Lower Jurassic. Sedimentology.

[CR69] Jenny D, Fuchs D, Arkhipkin AI, Hauff RB, Fritschi B, Klug C (2019). Predatory behavior and taphonomy of a Jurassic belemnoid coleoid (Diplobelida, Cephalopoda). Scientific Reports.

[CR70] Jereb, P., Roper, C. F. E., Norman, M. D. & Finn, J. K. (eds., 2014). *Cephalopods of the World: An Annotated and Illustrated Catalogue of Cephalopod Species Known to Date. 3: Octopods and Vampire Squids.* Food and Agriculture Organization of the United Nations, Rome.

[CR71] Keupp H, Engeser T, Fuchs D, Haechel W (2010). Ein *Trachyteuthis hastiformis* (Cephalopoda, Coleoidea) mit Spermatophoren aus dem Ober-Kimmeridgium von Painten (Ostbayern). Archaeopteryx.

[CR72] Klug C, Di Silvestro G, Hoffmann R, Schweigert G, Fuchs D, Clements T, Guériau P (2021). Diagenetic phosphatic Liesegang rings deceptively resemble chromatophores in Mesozoic coleoids. PeerJ.

[CR75] Klug C, Frey L, Korn D, Jattiot R, Rücklin M (2016). The oldest Gondwanan cephalopod mandibles (Hangenberg Black Shale, Late Devonian) and the Mid-Palaeozoic rise of jaws. Palaeontology.

[CR76] Klug C, Fuchs D, Schweigert G, Röper M, Tischlinger H (2015). New anatomical information on arms and fins from exceptionally preserved *Plesioteuthis* (Coleoidea) from the Late Jurassic of Germany. Swiss Journal of Palaeontology.

[CR77] Klug C, Landman NH, Fuchs D, Mapes RH, Pohle A, Gueriau P, Reguer S, Hoffmann R (2019). Anatomy of the first Coleoidea and character evolution in the Carboniferous. Communications Biology.

[CR78] Klug C, Schweigert G, Dietl G (2010). A new *Plesioteuthis* with beak from the Kimmeridgian of Nusplingen (Germany). In: Fuchs, D. (Ed.), Proceedings of the Third International Coleoid Symposium. Ferrantia.

[CR79] Klug C, Schweigert G, Fuchs D, Kruta I, Tischlinger H (2016). Adaptations to squid-style high-speed swimming in Jurassic belemnitids. Biology Letters.

[CR80] Klug C, Schweigert G, Dietl G, Fuchs D (2005). Coleoid beaks from the Nusplingen Lithographic Limestone (Late Kimmeridgian, SW Germany). Lethaia.

[CR81] Košťák M, Schlögl J, Fuchs D, Holcová K, Hudáčková N, Culka A, Fözy I, Tomašových A, Milovský R, Šurka J, Mazuch M (2021). Fossil evidence for vampire squid inhabiting oxygen-depleted ocean zones since at least the Oligocene. Communications Biology.

[CR82] Kröger B, Vinther J, Fuchs D (2011). Cephalopod origin and evolution: a congruent picture emerging from fossils, development and molecules. BioEssays.

[CR83] Kruta I, Rouget I, Charbonnier S, Bardin J, Fernandez V, Germain D, Brayard A, Landman NH (2016). *Proteroctopus ribeti* in coleoid evolution. Palaeontology.

[CR84] Landman NL, Davis RA (1988). Jaw and crop preserved in an orthoconic nautiloid cephalopod from the Bear Gulch Limestone (Mississippian, Montana). New Mexico Bureau of Mines and Mineral Resources.

[CR85] Lindgren AR, Giribet G, Nishigushi MK (2004). A combined approach to the phylogeny of Cephalopoda (Mollusca). Cladistics.

[CR86] Lordan C, Collins MA, Perales-Raya C (1998). Observations on morphology, age and diet of three *Architeuthis* caught off the west coast of Ireland in 1995. Marine Biologists’ Association UK.

[CR87] Mapes RH, Landman NH, Klug C (2019). Caught in the act? Distraction sinking in ammonoid cephalopods. Swiss Journal of Palaeontology.

[CR88] Mapes RH, Weller EA, Doguzhaeva LA, Tanabe K, Shigeta Y, Sasaki T, Hirano H (2010). Cephalopods showing a tentacle with arm hooks and an ink sac from Montana, USA. Cephalopods - Present and Past.

[CR89] Müller H (1853). Ueber das Männchen von *Argonauta Argo* und die Hectocotylen. Zeitschrift für wissenschaftliche Zoologie.

[CR90] Münster GG (1843). Die schalenlosen Cephalopoden im unteren Jura, den Lias-Schiefern von Franken und Schwaben. Beiträge zur Petrefaktenkunde.

[CR91] Naef A (1922). Die fossilen Tintenfische.

[CR92] Nixon M (1985). Capture of prey, diet and feeding of *Sepia officinalis* and *Octopus vulgaris* (Mollusca: Cephalopoda) from hatchling to adult. Vie et milieu.

[CR93] Nixon M, Boyle PR (1987). The diets of cephalopods. Cephalopod life cycles, 2.

[CR94] Nixon M, Wiedmann J, Kullmann J (1988). The feeding mechanisms and diets of cephalopods - living and fossil. Cephalopods - Present and Past.

[CR96] Palacio FJ (1973). On the double hectocotylization of octopods. The Nautilus.

[CR97] Přikryl T, Košt’ák, M., Mazuch, M., & Mikuláš, R. (2012). Evidence for fish predation on a coleoid cephalopod from the Lower Jurassic Posidonia Shale of Germany. Neues Jahrbuch für Geologie und Paläontologie, Abhandlungen.

[CR98] Quenstedt, F. A. (1845–49). *Petrefactenkunde Deutschlands, 1. Abteilung, 1. Band, Cephalopoden*. Tübingen, Verlag Fues.

[CR99] Radwański A, Kin A, Radwańska U (2009). Queues of blind phacopid trilobites Trimerocephalus: A case of frozen behaviour of Early Famennian age from the Holy Cross Mountains, Central Poland. Acta Geologica Polonica.

[CR100] Riegraf W, Werner G, Lörcher F (1984). Der Posidonienschiefer.

[CR101] Robson GC (1929). On a case of bilateral hectocotylization in *Octopus rugosus*. Journal of Zoology.

[CR102] Röhl A, Schmid-Röhl HJ, Oschmann W, Frimmel A, Schwark L (2002). Palaeoenvironmental reconstruction of Lower Toarcian epicontinental black shales (Posidonia Shale, SW Germany): Global versus regional control. Geobios.

[CR103] Röhl HJ, Schmid-Röhl A, Oschmann W, Frimmel A, Schwark L (2001). The Posidonia Shale (Lower Toarcian) of SW-Germany: An oxygen-depleted ecosystem controlled by sea level and palaeoclimate. Palaeogeography, Palaeoclimatology, Palaeoecology.

[CR104] Rosa R, Lopes VM, Guerreiro M, Bolstad K, Xavier JC (2017). Biology and ecology of the world’s largest invertebrate, the colossal squid (*Mesonychoteuthis hamiltoni*): a short review. Polar Biology.

[CR105] Seilacher A (1970). Begriff und Bedeutung der Fossil-Lagerstätten. Neues Jahrbuch für Geologie und Paläontologie, Monatshefte.

[CR106] Strugnell J, Jackson J, Drummond AJ, Cooper AA (2006). Divergence time estimates for major cephalopod groups: Evidence from multiple genes. Cladistics.

[CR111] Villanueva R, Perricone V, Fiorito G (2017). Cephalopods as predators: A short journey among behavioral flexibilities, adaptions, and feeding habits. Frontiers in Physiology.

[CR112] Voight JR (1997). Cladistic analysis of the octopods based on anatomical characters. Journal of Molluscan Studies.

[CR113] Wilby P, Hudson JD, Clements RG, Hollingworth NTJ (2004). Taphonomy and origin of an accumulate of soft-bodied cephalopods in the Oxford Clay Formation (Jurassic England). Palaeontology.

[CR114] Ylitalo H, Oliver TA, Fernandez-Silva I, Wood JB, Toonen RJ (2019). A behavioral and genetic study of multiple paternity in a polygamous marine invertebrate *Octopus oliveri*. PeerJ.

[CR115] Young RE, Vecchione M, Donovan DT (1998). The Evolution of Cephalopods and their present Biodiversity and Ecology. South Africa Journal of Marine Science.

